# The impact of limb loading and the measurement modality (2D versus 3D) on the measurement of the limb loading dependent lower extremity parameters

**DOI:** 10.1186/s12891-020-03449-1

**Published:** 2020-06-30

**Authors:** Lukas Jud, Tabitha Roth, Philipp Fürnstahl, Lazaros Vlachopoulos, Reto Sutter, Sandro F. Fucentese

**Affiliations:** 1 Department of Orthopedics, Balgrist University Hospital, University of Zurich, Forchstrasse 340, 8008 Zürich, Switzerland; 2 Institute for Biomechanics, ETH Zurich, Zurich, Switzerland; 3 Research in Orthopedic Computer Science (ROCS), Balgrist University Hospital, University of Zurich, Zurich, Switzerland; 4 Department of Radiology, Balgrist University Hospital, University of Zurich, Forchstrasse 340, 8008 Zurich, Switzerland

**Keywords:** Three-dimensional planning, Navigation, Corrective osteotomy, Osteotomy, Computer-assisted surgery, Leg deformity

## Abstract

**Background:**

Deformity assessment and preoperative planning of realignment surgery are conventionally based on weight-bearing (WB) radiographs. However, newer technologies such as three-dimensional (3D) preoperative planning and surgical navigation with patient-specific instruments (PSI) rely on non-weight bearing (NWB) computed tomography (CT) data. Additionally, differences between conventional two-dimensional (2D) and 3D measurements are known. The goal of the present study was to systematically analyse the influence of WB and the measurement modality (2D versus 3D) on common WB-dependent measurements used for deformity assessment.

**Methods:**

85 lower limbs could be included. Two readers measured the hip-knee-ankle angle (HKA) and the joint line convergence angle (JLCA) in 2D WB and 2D NWB radiographs, as well as in CT-reconstructed 3D models using an already established 3D measurement method for HKA, and a newly developed 3D measurement method for JLCA, respectively. Interrater and intermodality reliability was assessed.

**Results:**

Significant differences between WB and NWB measurements were found for HKA (*p* < 0.001) and JLCA (*p* < 0.001). No significant difference could be observed between 2D HKA NWB and 3D HKA (*p* = 0.09). The difference between 2D JLCA NWB and 3D JLCA was significant (*p* < 0.001). The intraclass correlation coefficient (ICC) for the interrater agreement was almost perfect for all HKA and 3D JLCA measurements and substantial for 2D JLCA WB and 2D JLCA NWB. ICC for the intermodality agreement was almost perfect between 2D HKA WB and 2D HKA NWB as well as between 2D HKA NWB and 3D HKA, whereas it was moderate between 2D JLCA WB and 2D JLCA NWB and between 2D JLCA NWB and 3D JLCA.

**Conclusion:**

Limb loading results in significant differences for both HKA and JLCA measurements. Furthermore, 2D projections were found to be insufficient to represent 3D joint anatomy in complex cases. With an increasing number of surgical approaches based on NWB CT-reconstructed models, research should focus on the development of 3D planning methods that consider the effects of WB on leg alignment.

## Background

Preoperative planning for realignment surgery of the lower extremity is conventionally based on weight-bearing (WB) radiographs [27]. However, in the last years, approaches based on computed tomography- (CT) reconstructed three-dimensional (3D) models have been increasingly established [5, 8, 10, 15]. Likewise, an increasing publication trend towards preoperative 3D planning in orthopaedic surgery can be observed in the current literature [33]. They provide the surgeon with an improved understanding of the deformity, enable more sophisticated preoperative planning, and support the surgeon in the precise execution of the preoperative plan through surgical navigation [15, 16]. One example of surgical navigation is the use of patient-specific instruments (PSI), which are well established in realignment surgery such as knee arthroplasty, high tibial osteotomy, or opening- and closing-wedge distal femoral osteotomies [6, 12, 25, 31]. All these approaches rely on CT-based 3D reconstructions. However, CT is commonly acquired in a supine position and, therefore, is lacking information about the effect of WB on leg alignment.

Deformity assessment in the state-of-the-art computer-assisted planning is performed in 3D. Although 3D measurements show low intra- and interobserver variability [9], differences between two-dimensional (2D) and 3D measurements of the hip-knee-ankle angle (HKA) have been shown [19]. Thus, considerable measurement errors have to be expected in preoperative 3D planning as a result of the discrepancies between the WB and non-weight-bearing (NWB) conditions as well as due to the used measurement modality itself. The goal of this study was to perform a systematic analysis of the impact of limb loading (WB versus NWB) and the measurement modality (2D versus 3D) on common limb loading-dependent lower extremity parameters. As the joint line convergence angle (JLCA) was shown to be the most important preoperative factor that predicts realignment discrepancy after high tibial osteotomy [32], JLCA was investigated as the second parameter besides the most used parameter in realignment surgery in daily practice, the HKA. We hypothesize that HKA and JLCA measurements are significantly different depending on limb loading (WB versus NWB) and the applied measurement modality (2D versus 3D).

## Methods

The local ethical committee approved this study (Zurich Cantonal Ethics Commission, KEK 2018–02242) and all patients gave their written informed consent.

### Patient selection and imaging

All patients that were subject to a corrective osteotomy of the distal femur and/or the proximal tibia between April 2016 and January 2019 at our institution were included. 91 patients could be identified. The exclusion criterion was an incomplete radiological dataset; therefore, nine patients had to be excluded. Three of the included patients underwent surgery on both legs, resulting in a total of 85 legs. The mean age at the time of surgery was 40.4 ± 11.3 years (range 15–64). 74.1% (*n* = 63) of the patients were males and 52.9% (*n* = 45) were left legs. Two independent readers (JL, RT) performed all measurements using preoperative radiological data.

The full preoperative radiological dataset contained the following:

- A standing long-leg radiograph (EOS imaging system, EOS, Paris, France) representing 2D WB imaging. The radiographs were acquired in WB upright standing position, the knee in full extension, and the patella facing directly forwards. The X-ray beam path was orientated parallel to the joint line of the knee.
- A CT scan (Philips Brilliance 64, Philips Healthcare, Best, The Netherlands, or Somatom Definition AS Siemens Healthcare, Erlangen, Germany) of the affected lower extremity representing 3D NWB imaging. The data acquisition was performed using the MyOsteotomy CT protocol (Medacta SA, Switzerland) in which only the hip, knee, and ankle joints were scanned, while skipping irrelevant midshaft regions. Slice thickness was 1.0 mm with an in-plane resolution of 0.4 × 0.4 mm. CT scans were acquired with the patients in a supine position and the knee in full extension.
- The CT scan included a supine position scanogram, which was used for 2D NWB measurements.

### 2D measurements

**Fig. 1:**
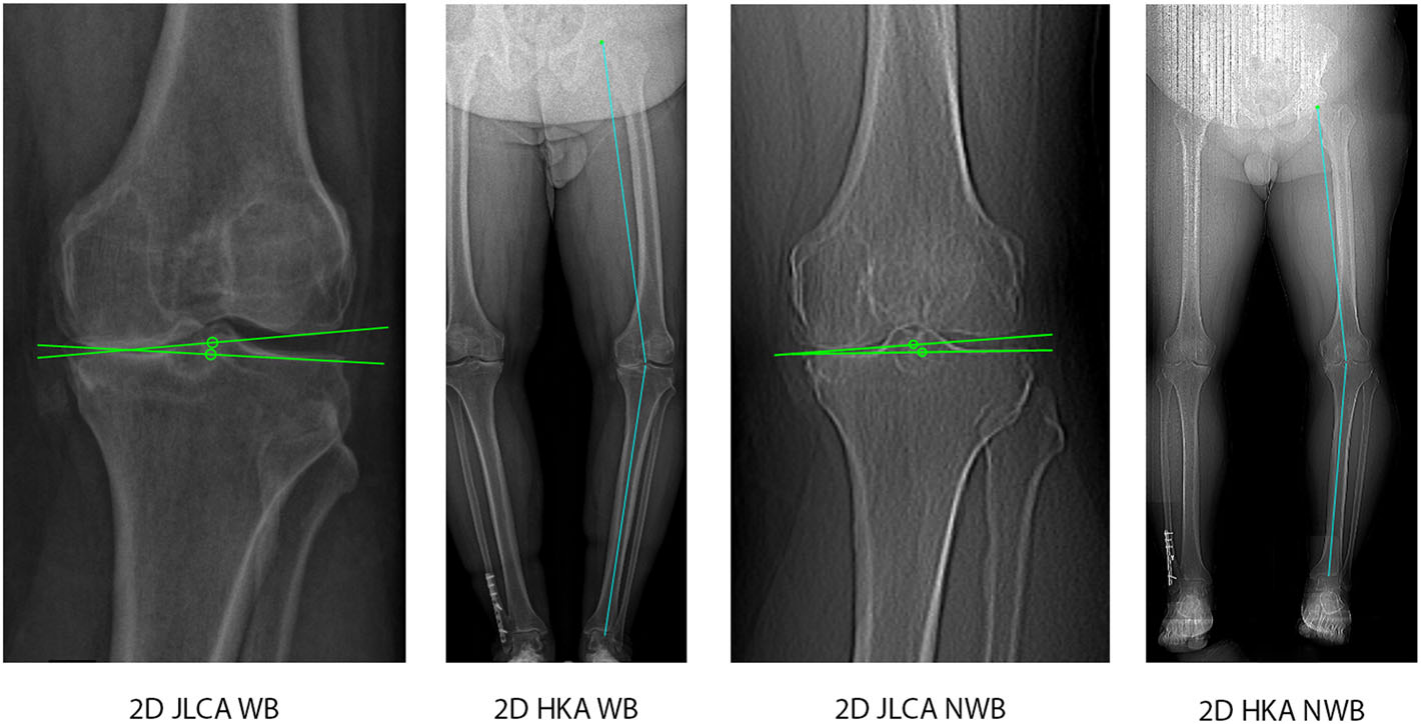
Measurements of 2D HKA and 2D JLCA in WB and NWB conditions. On the left side, measurements were performed using the long-leg radiographs (i.e., WB condition). On the right side, measurements were performed using the scanogram (i.e., NWB condition)

The mechanical axis, denoted as 2D HKA, and the 2D JLCA were measured on standing long-leg radiographs (WB) and the scanograms (NWB) using the Long Leg Osteotomy module of mediCAD (version 5.1.0.7, mediCAD Hectec GmbH, Altdorf, Germany). Both measurements were performed according to the measurement methods described by Paley [26] (Fig. 1). The hip joint centre (HJC) was defined as the centre of a circle fitted to the femoral head. Femoral and tibial knee bases were separately determined by drawing two baseline tangents to the corresponding articular surfaces. The femoral and tibial knee joint centres (fKJC and tKJC, respectively) were defined on the baseline tangents and midway between the lateral and medial femoral condyles and the lateral and medial tibial plateau, respectively. Similarly, the ankle joint base was determined by drawing a tangent to the articular surface of the talus. The ankle joint centre (AJC) was determined on the talus tangent and midway between two parallel lines on the lateral and medial talus. Subsequently, the HKA angle was measured as the angle between the lines connecting the HJC and the fKJC and the line connecting the tKJC to the AJC. A varus angle was denoted as a positive value, and a valgus angle as a negative value.

The JLCA was measured as the angle between the femoral and tibial baseline tangents, whereby laterally opened angles were interpreted as positive values and medially opened angles as negative values.

### 3D measurements

**Fig. 2:**
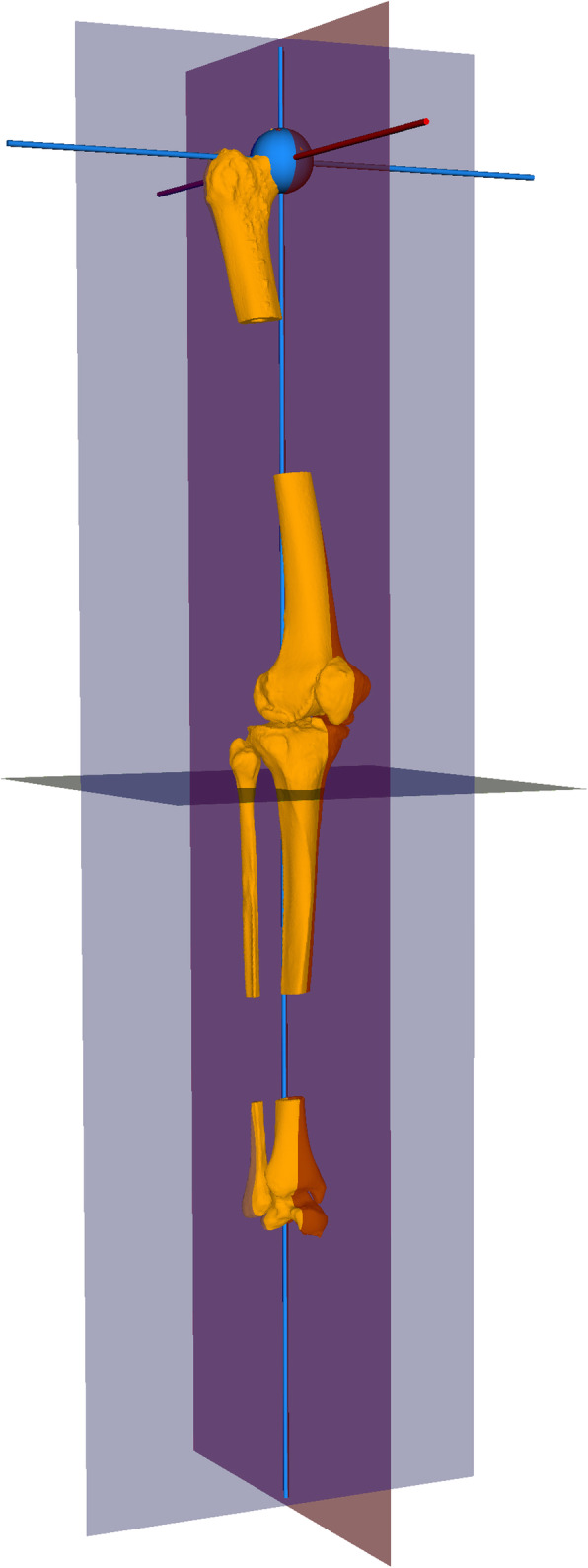
Alignment of the 3D bone models. The leg models were reorientated within the reference coordinate system of CASPA, in a way that the mechanical leg axis was aligned with the y-axis of the coordinate system (normal of axial plane) and the patella was in line with the z-axis (normal of frontal plane)

3D bone models of all included legs (proximal and distal femur, tibia, and fibula, as well as patella and talus) were generated from CT data using commercial segmentation software (Mimics Medical 19.0, Materialise NV, Leuven, Belgium). The 3D deformity measurements were carried out using a combination of the 3D preoperative planning software CASPA (version 5.29, Balgrist CARD, Zurich, Switzerland) and MATLAB (version 2019a, The MathWorks Inc., Natick, MA, USA). In a first step, the leg models were reorientated within the reference coordinate system of CASPA, aligning the mechanical leg axis with the y-axis (perpendicular to the axial plane). The models were then rotated around this axis until the anterior surface of the patella was aligned with the z-axis (perpendicular to the frontal plane) of the coordinate system (Fig. 2).

**Fig. 3:**
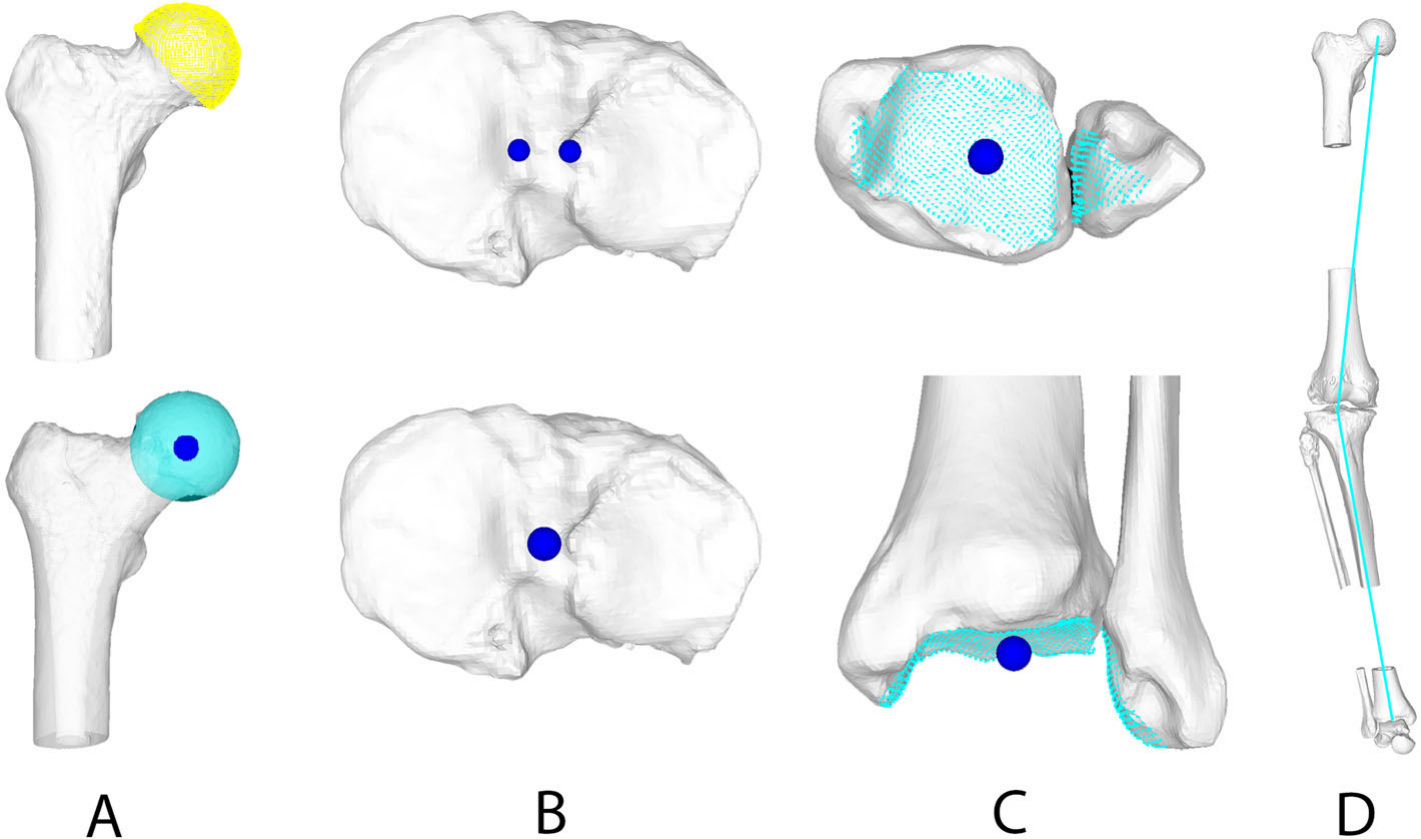
Measurement of 3D HKA. **a** A sphere was fitted to the femoral head to determine the hip joint centre. **b** The knee joint centre was located midway between the tibial eminences. **c** The ankle joint centre was calculated as the centre of mass of all points of the articular surface of the distal tibia and fibula. **d** Schematic marked the measurement of 3D HKA. A line connecting the HJC and the KJC and a second line connecting the KJC and the AJC were projected to the frontal plane to measure 3D HKA

The 3D HKA was measured using the same methodology as described by Fürnstahl et al. [10], and already used and validated in different other studies [13, 14, 16]. The HJC was defined as the centre of a sphere, fitted to the femoral head by using least-square regression [29], minimizing the distance to a user-selected region of the femoral head. Following Moreland et al. [23], the KJC was defined as the midpoint between the intercondylar eminences on the tibial plateau. Lastly, the AJC was determined as the centre of the distal articular surface of the tibia and fibula with respect to the lateral and medial malleolus. To this end, the articular surface was determined by registering all points on the articular surface of the distal tibia and fibula within a certain user-defined distance (typically 3–5 mm) to the talus, using the closest-point distance [24]. The AJC was then computed as the centre of mass of all selected points on the articular surface of the distal tibia and fibula. Finally, the 3D HKA was calculated as the angle between a line connecting the HJC and the KJC and a second line between the KJC and the AJC, projected on the frontal plane (Fig. 3).

**Fig. 4:**
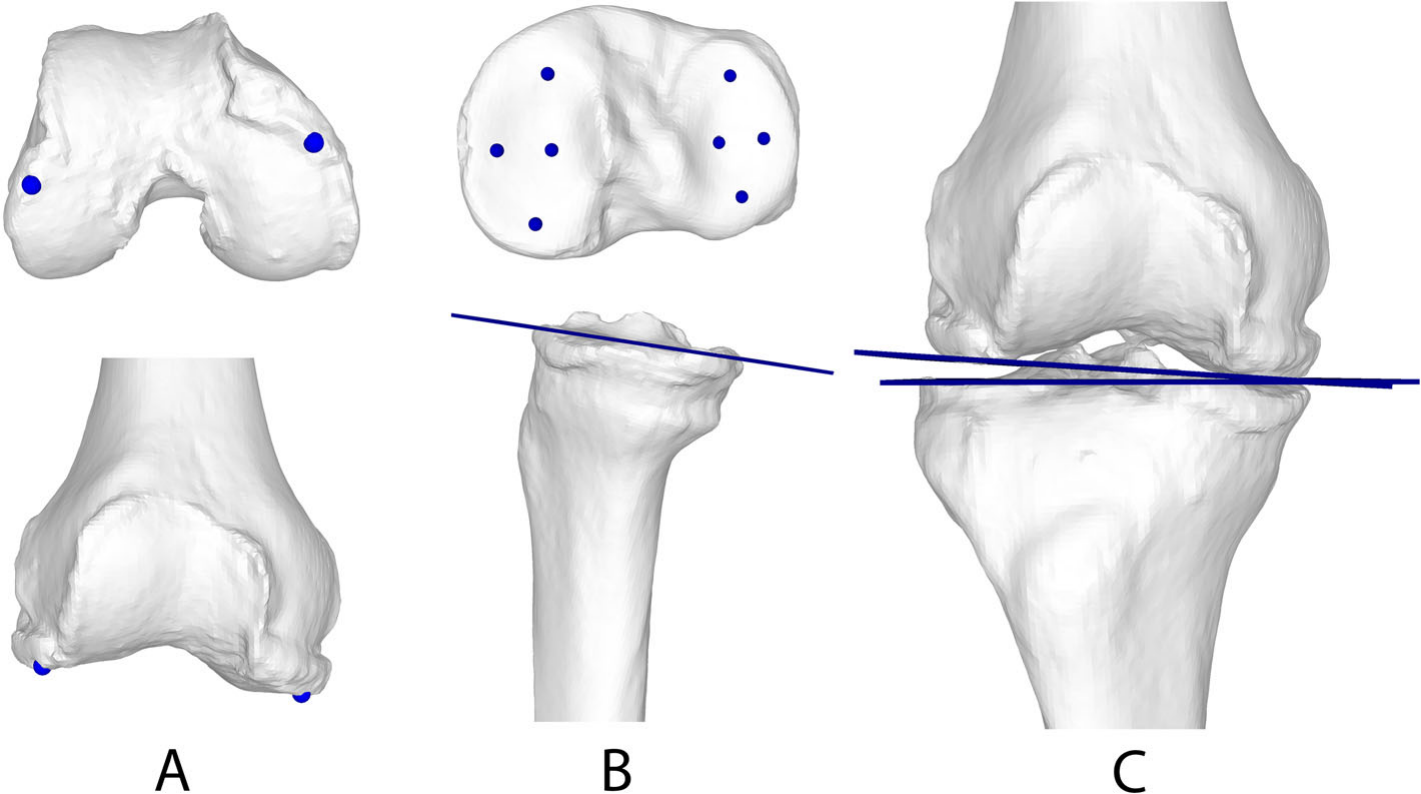
Measurement of 3D JLCA. **a** The most distal points of the femur were connected. **b** A plane was fitted to the tibial plateau by selecting four points on the medial and four points on the lateral tibial plateau. **c** The femoral condyle tangent and the tibial condyle tangent were projected to the frontal plane to measure the 3D JLCA

For measuring the 3D JLCA, the longitudinal axis of the femur was determined using principal component analysis (PCA), which minimises the squared distances of the proposed axis to all points of the femur [29]. Subsequently, the medial and lateral femoral condyles were automatically clustered by applying a k-means algorithm [7] to the distal femoral epiphysis, initialised as described by Arthur et al. [1]. The three most distal points along the longitudinal axis were then defined for each of the two clustered condyles, and their means were used to define the femoral condyle tangent (FCT). The tibial condyle tangent (TCT) was defined as the frontal projection of the tibial plateau plane. A least squares approach [29] was used to determine the tibial plateau plane, minimising the distance to user-selected four points on the medial and four points on the lateral tibial plateau. Finally, the 3D JLCA was calculated as the angle between the FCT and the TCT (Fig. 4).

### Statistical analysis

Measurements were analysed using SPSS (IBM SPSS for Windows, version 26, Armonk, NY, USA). Means were reported as mean standard deviation (SD). Interrater agreement was calculated using the two-way random intraclass correlation coefficient (ICC) for the absolute agreement of single measures. The correlation between methods was determined using the ICC two-way mixed model for the absolute agreement of average measures (average values of both raters were used). The correlation between the effect of WB on HKA and JLCA was assessed by calculating the ICC (two-way random model for consistency of rater averaged measures) for the differences between 2D WB and 2D NWB measures. Intraclass correlation coefficients were interpreted according to Landis and Koch [18]. Therefore, ICC’s between 0.81 and 1.00 were interpreted as almost perfect, between 0.61 and 0.80 as substantial, between 0.41 and 0.60 as moderate, between 0.21 and 0.40 as fair, between 0.00 and 0.20 as slight, and < 0.00 as poor. For intermethod analyses, differences of means between raters were compared using paired Student’s *t*-tests. Mean absolute differences (MAD) between raters as well as between rater averaged results of different modalities were calculated. Tests were evaluated using a significance level of p ≤ 0.05.

## Results

### Interrater agreement

An overview of the measurements and the interrater agreement is given in Table 1. The ICC for interrater agreement was almost perfect for 2D HKA WB [0.996 (95%CI: 0.994–0.998)], 2D HKA NWB [0.987 (95%CI: 0.980–0.991)], 3D HKA [0.988 (95%CI: 0.981–0.992)], and 3D JLCA [0.844 (95%CI: 0.743–0.903)]. It was substantial for 2D JLCA WB [0.783 (95%CI: 0.684–0.853)] and 2D JLCA NWB [0.694 (95%CI, 0.563–0.790)].

**Table 1:** Overview of interrater agreement and reader analysis

**INTERRATER AGREEMENT ICC**			
	**2D WB**	**2D NWB**	**3D**
HKA (95% CI)	0.996 (0.994–0.998)	0.987 (0.980–0.991)	0.988 (0.981–0.992)
JLCA (95% CI)	0.783 (0.684–0.853)	0.694 (0.563–0.790)	0.844 (0.743–0.903)
**READER ANALYSIS**			
	**2D WB**	**2D NWB**	**3D**
**HKA**			
Reader 1: Mean (range)	5.4 (−10.5–19.5)	4.4 (6.3–15.3)	4.2 (− 7.2–19.8)
Reader 2: Mean (range)	5.5 (−10.9–19.2)	4.5 (− 5.7–14.8)	4.3 (−8.2–19.6)
MAD: Mean ± SD	0.3 ± 0.3	0.4 ± 0.4	0.4 ± 0.5
**JLCA**			
Reader 1: Mean (range)	2.2 (−9.8–12.6)	1.2 (− 5.6–7.0)	3.3 (−6.0–10.6)
Reader 2: Mean (range)	2.2 (− 5.3–9.7)	1.2 (− 3.9–5.9)	3.8 (−4.0–9.5)
MAD: Mean ± SD	1.3 ± 1.2	1.2 ± 1.0	0.9 ± 0.9

Intraclass correlation coefficient (*ICC*), two-dimensional (*2D*), three-dimensional (*3D*), weight-bearing (*WB*), non-weight-bearing (*NWB*), hip-knee-ankle angle (*HKA*), joint line convergence angle (*JLCA*), confidence interval (*CI*), mean absolute difference (*MAD*), and standard deviation (*SD*)

### Intermodality agreement

An overview of the intermodality agreement is given in Table 2. 2D HKA WB and 2D HKA NWB were significantly different (5.5° ± 4.8 versus 4.5° ± 3.6, *p* < 0.001, MAD = 1.7 ± 1.3° (0–5.2)). No significant difference could be observed between 2D HKA NWB and 3D HKA (4.5° ± 3.6 versus 4.2° ± 4.0, *p* = 0.09, MAD = 1.1 ± 1.0° (0–5.0)).

**Table 2:** Overview of intermodality agreement

INTERMODALITY AGREEMENT ICC		
	2D WB versus 2D NWB	2D NWB versus 3D
**HKA**		
ICC (95% CI)	0.937 (0.866–0.966)	0.968 (0.950–0.979)
MAD: Mean ± SD	1.7 ± 1.3 (0–5.2)	1.1 ± 1.0 (0–5.0)
T-Test (Mean ± SD (p-value))	5.5° ± 4.8 versus 4.5° ± 3.6 (p < 0.001)	4.5° ± 3.6 versus 4.2° ± 4.0 (p = 0.09)
**JLCA**		
ICC (95% CI)	0.520 (0.253–0.690)	0.498 (− 0.166–0.764)
MAD: Mean ± SD	1.9 ± 1.9 (0–14.0)	2.5 ± 1.7 (0.2–9.1)
T-Test (Mean ± SD (*p*-value))	2.2° ± 2.5 versus 1.2° ± 1.9 (p < 0.001)	1.2° ± 1.9 versus 3.5° ± 2.2 (p < 0.001)

Intraclass correlation coefficient (*ICC*), two-dimensional (*2D*), three-dimensional (*3D*), weight-bearing (*WB*), non-weight-bearing (*NWB*), hip-knee-ankle angle (*HKA*), joint line convergence angle (*JLCA*), confidence interval (*CI*), mean absolute difference (*MAD*), and standard deviation (*SD*)

2D JLCA WB and 2D JLCA NWB were shown to be significantly different (2.2° ± 2.5 versus 1.2° ± 1.9, *p* < 0.001, MAD = 1.9 ± 1.9° (0–14.0)), as well as 2D JLCA NWB and 3D JLCA (1.2° ± 1.9 versus 3.5° ± 2.2, *p* < 0.001, MAD = 2.5 ± 1.7° (0.2–9.1)).

ICC for intermodality agreement was almost perfect between 2D HKA WB and 2D HKA NWB [0. 937 (95%CI: 0.866–0.966)] as well as 2D HKA NWB and 3D HKA [0. 968 (95%CI: 0.950–0.979)]. It was moderate for 2D JLCA WB and 2D JLCA NWB [0. 520 (95%CI: 0.253–0.690)] and between 2D JLCA NWB and 3D JLCA [0. 498 (95%CI: − 0.166 – 0.764)].

### Correlation between WB effects on HKA and JLCA

The differences between 2D WB and 2D NWB measures for HKA and JLCA, respectively, showed a substantial ICC of 0.717 (95%CI: 0.565–0.816).

## Discussion

The most important finding of this study is that both WB and 3D anatomy are important factors that must be considered when planning corrective osteotomies in the future. HKA and JLCA were significantly different when weight was applied, and, therefore, the hypothesis regarding limb loading could be confirmed. Regarding the hypothesis that also the applied measurement modality results in marked differences, this could only be partially confirmed. While the modality (2D versus 3D) did not markedly influence the measures for HKA, JLCA measures were correlated only moderately, therefore, indicating the need for a more detailed assessment, as it is possible by considering the 3D anatomy using reconstructed 3D models. Regarding clinically meaningful measurement differences for HKA, a threshold value of 2° might be considered as reasonable, as the accuracy of realignment surgery (i.e., high tibial osteotomy) is reported to be 2° [30]. For JLCA measurements, the same threshold value of 2° can be considered as reasonable, as a substantial correlation could be demonstrated in this study between WB effects on HKA and JLCA and no such threshold values for JLCA are reported in the current literature so far. Regarding the intermodality agreement, the threshold value of 2° was exceeded between all measurement modalities (MAD plus SD) in this study, further indicating the importance of a detailed assessment.

**Fig. 5:**
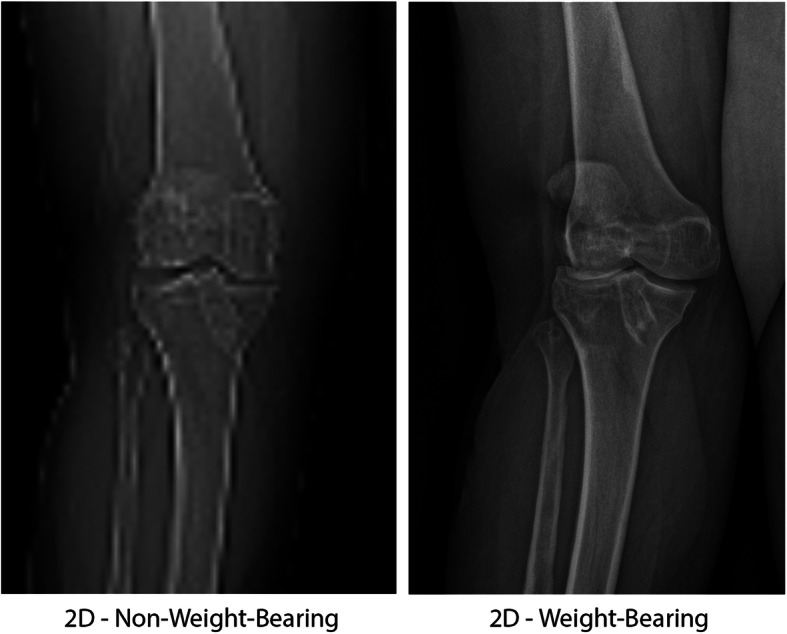
Substantial discrepancies between knee joint alignment of the same patient in NWB (left) and WB (right) conditions

Previous studies have already shown discrepancies between the measurements of HKA in WB and NWB conditions [4, 28]. In line with our results, Brouwer et al. found a mean difference of 2° in standing and supine radiographs of 20 varus knees [4]. The same applies to Paternostre et al. who described a difference of 2° or more in 46% of their 70 included patients [28]. The effect of WB on the JLCA was less frequently investigated so far. However, So et al. showed a mean difference of 1.8° between WB and NWB conditions for the measurement of JLCA [32], which is comparable to the results of this study. At the same time, they found it to be the most important predicting factor for coronal correction discrepancy after medial opening-wedge high tibial osteotomy. Similarly, Lee et al. reported that the change in JLCA between pre- and postoperative measures significantly correlates with the correction error [20]. According to them, JLCA can be seen as an indicator for ligament laxity, and WB is required to assess the degree of stability provided by them. In line with that, the correlation of 2D JLCA WB and 2D JLCA NWB in this study was only moderate and 15% of all patients had differences of more than 3°. As depicted in Fig. 5, these differences can lead to a considerably different impression of the preoperative anatomy and can, therefore, substantially influence the surgeons’ decision.

**Fig. 6:**
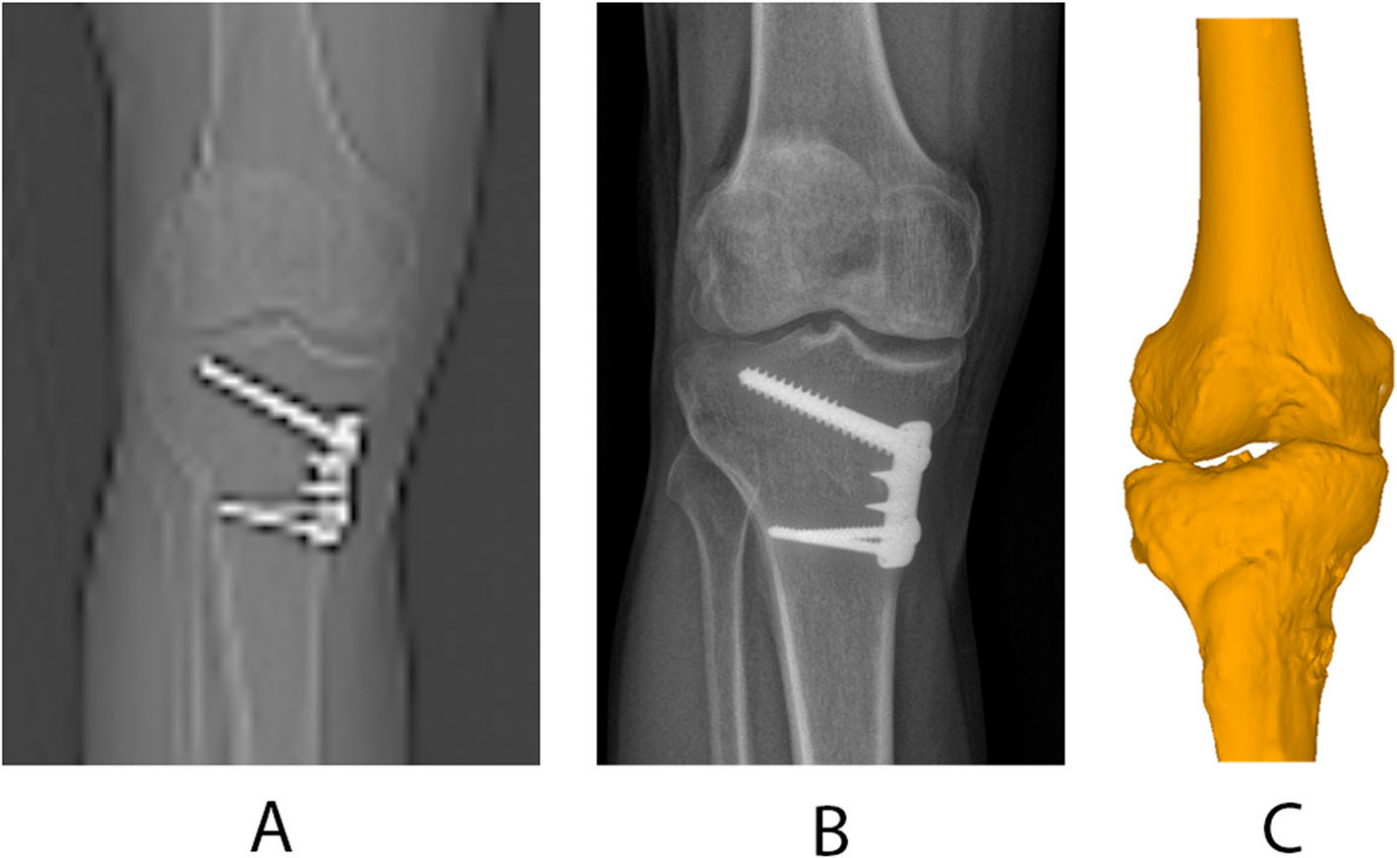
Substantial differences in the depiction of the tibial plateau between 2D and 3D. While the 3D model **c** can accurately represent the patients’ anatomy, the 2D projections (scanogram **a** and radiograph **b**) do not adequately display the full information

However, adhering to the conventional 2D methods does not seem to be an option either. The correlation between 2D JLCA NWB and 3D JLCA was moderate only, and 15% of the patients had a MAD of 4.1° or more between the 3D anatomy (as represented by the CT-reconstructed 3D model) and the depicted projections in 2D. This was true for both the scanogram and the radiograph (Fig. 6). As low sensitivity for the measurement of the knee joint space width in conventional radiographs is known and CT or MRI is supposed to be more accurate [17, 21, 34], the findings of this study suggest that 2D imaging modalities are not sufficient to assess 3D anatomy.

The preferable use of the 3D JLCA measurement is further strengthened by the almost perfect interrater agreement, compared to the only substantial agreement for the 2D JLCA measurements, and therefore, indicates more reliable measurements using 3D JLCA. Likewise, Furmetz et al. demonstrated low intra- and interobserver variability in 3D measurements of the lower extremity parameters [9], encouraging the application of 3D measurements in this area. If measured in NWB images, 3D HKA measurements in this study correlated very well with the conventional 2D method. This is in agreement with Lazennec et al., who found no significant difference between 2D biplanar radiographs and their corresponding 3D models [19].

The use of 3D measurement techniques seems indispensable, as an increasing number of surgical techniques, based on CT-reconstructed 3D models, are getting established [5, 8, 10, 15]. Besides an improved understanding of the deformities, it could be shown that preoperative 3D planning in combination with navigation-aids results in more accurate deformity correction in realignment surgery compared to conventional techniques [12].

Besides the need for 3D preoperative planning, it is important to incorporate WB into the surgical planning workflow, not to neglect the resulting differences in measuring lower limb parameters caused by limb loading [4, 28, 32]. Correctly estimating the HKA is mandatory, as precise axis correction in coronal realignment surgery is crucial for a successful postoperative result, and under- or overcorrection, probably partly as a result of the measurement discrepancies, was shown to be the main reason for clinical failure [3, 11]. However, as WB is important, the next step should be the integration of 2D/3D registration into preoperative planning. Furthermore, patient-specific simulations, including simulation of WB, could further improve the planning of realignment surgery.

A limitation of this study is that the 2D NWB measurements were performed using the CT scanogram. These images have a low resolution and are bearing the risk that the leg was not orientated with the patella facing directly forwards, as it is requested for correct acquisition of long-leg radiographs [2]. However, it could be shown that isolated rotation of the leg has no relevant influence on HKA measurements [14]. Furthermore, it could be demonstrated that the use of a CT scanogram for measuring HKA shows reasonable accuracy [22].

## Conclusion

Significant differences in HKA as well as JLCA could be observed upon limb loading, which emphasizes the need for consideration of WB in the planning process of realignment surgery. Besides, 2D projected imaging methods were found to be insufficient to assess the JLCA in some patients, underlining the need for 3D planning methods. Together with an increasing number of surgical approaches based on CT-reconstructed 3D models, this points out the need for further research directed towards patient-specific 3D planning with the possibility of incorporating WB effects.

## Data Availability

Anonymized source data can be obtained from the corresponding author on reasonable request.

## Abbreviations

WB: Weight-bearing
3D: Three-dimensional
PSI: Patient-specific instruments
NWB: Non-weight bearing
CT: Computed-tomography
2D: Two-dimensional
HKA: Hip-knee-ankle angle
JLCA: Joint line convergence angle
HJC: Hip joint centre
fKJC: Femoral knee joint centre
tKJC: Tibial knee joint centre
AJC: Ankle joint centre
FCT: Femoral condyle tangent
TCT: Tibial condyle tangent
MAD: Mean absolute difference
ICC: Intraclass correlation coefficient
